# Global translational induction during NLR-mediated immunity in plants is dynamically regulated by CDC123, an ATP-sensitive protein

**DOI:** 10.1016/j.chom.2023.01.014

**Published:** 2023-02-17

**Authors:** Tianyuan Chen, Guoyong Xu, Rui Mou, George H. Greene, Lijing Liu, Jonathan Motley, Xinnian Dong

**Affiliations:** 1 Howard Hughes Medical Institute, Department of Biology, Duke University, Durham, NC 27708, USA; 2 Present address: State Key Laboratory of Hybrid Rice, Institute for Advanced Studies (IAS), Wuhan University, Wuhan, Hubei 430072, China; 3 Present address: Upstream Biotechnology Inc., Durham, NC 27701, USA; 4 These authors contributed equally; 5 Lead contact

## Abstract

The recognition of pathogen effectors by their cognate nucleotide-binding leucine-rich repeat (NLR) receptors activates effector-triggered immunity (ETI) in plants. ETI is associated with correlated transcriptional and translational reprogramming and subsequent death of infected cells. Whether ETI-associated translation is actively regulated or passively driven by transcriptional dynamics remains unknown. In a genetic screen using a translational reporter, we identified CDC123, an ATP-grasp protein, as a key activator of ETI-associated translation and defense. During ETI, an increase in ATP concentration facilitates CDC123-mediated assembly of the eukaryotic translation initiation factor 2 (eIF2) complex. Because ATP is required for the activation of NLRs as well as the CDC123 function, we uncovered a possible mechanism by which the defense translatome is coordinately induced during NLR-mediated immunity. The conservation of the CDC123-mediated eIF2 assembly suggests its possible role in NLR-mediated immunity beyond plants.

## In brief

Chen et al. identify CDC123 as a key activator of protein translation during effector-triggered immunity (ETI) in plants. Elevated ATP concentration during ETI facilitates CDC123-mediated assembly of the eIF2 complex to promote translation. Conservation of CDC123-mediated eIF2 assembly suggests its possible role in NLR-mediated immunity beyond plants.

## Graphical Abstract

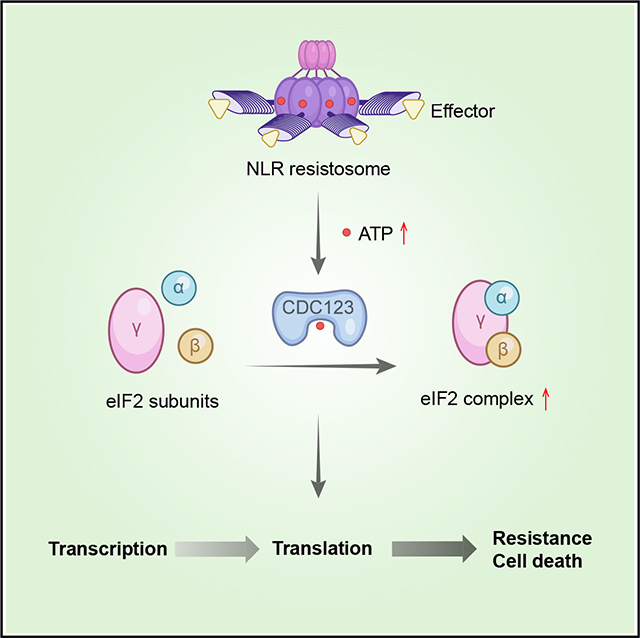

## INTRODUCTION

To recognize the presence of pathogen effectors, plants have evolved intracellular nucleotide-binding leucine-rich repeat (NLR) receptors to initiate effector-triggered immunity (ETI). When activated, NLRs undergo oligomerization through the exchange of ADP for ATP (or dATP) to form the resistosome, which triggers a Ca^2+^ influx^1–3^ and subsequent programmed cell death (PCD) to restrict pathogen proliferation. Interestingly, in contrast to apoptosis in animals, which involves an overall cessation in protein translation,^4^ ETI-associated cell death is preceded by dramatic transcriptional and translational reprogramming.^5–7^ Our previous study showed that these changes are highly correlated,^6^ raising the question of whether translation is actively regulated during this immune response or passively driven by transcript abundance.

The lack of an overall repression in translation during ETI suggests that if it is regulated, it is unlikely to be through the integrated stress response pathway well-studied in yeast and mammals, which involves phosphorylation of the α subunit of the eukaryotic translation initiation factor 2 (eIF2) complex.^8^ Stress-induced eIF2α phosphorylation inhibits the recycling of the eIF2 complex and consequently represses translation by slowing ribosome assembly.^9^ However, for stress-responsive transcripts containing upstream open reading frames (uORFs), a reduction in the available eIF2 complex allows the initiation complex to bypass the uORF inhibition and translate the main ORFs (mORFs).^10^ However, in plants, whether phosphorylation of eIF2α plays a role in ETI has not been tested genetically in the knockout mutant of *general control nonderepressible 2* (*GCN2*), which is the only kinase known to phosphorylate eIF2α.^11^

In this study, we found that upon ETI induction, there is a transient increase in global translational activity despite the GCN2-mediated phosphorylation of eIF2α. Using a genetic screen, we discovered that cell division cycle 123 (CDC123) (AT4G05440, also known as EDA35/BICE1), an ATP-grasp protein, plays an essential role in NLR-mediated translation and defense. Upon ETI induction, the assembly of the eIF2 complex, consisting of eIF2αβγ, is enhanced by CDC123 through the elevated ATP level, whereas a transient knockdown of the eIF2γ subunit blocks the onset of ETI. Therefore, our study identifies a mechanism in globally inducing defense protein production, which is required for NLR-mediated cell death and resistance.

## RESULTS

### ETI induction involves a major increase in protein synthesis

To monitor ETI-mediated translational dynamics, we introduced the translational reporter *35S:5′LS_TBF1_-FLUC* into both wild-type (WT) *Arabidopsis* and the *rps2* mutant defective in the coiled-coil (CC)-NLR receptor (CNL), Resistance to *P. syringae* 2 (RPS2), for the bacterial effector AvrRpt2.^12,13^ The transcription of *35S:5′LS_TBF1_-FLUC* is driven by the constitutive 35S promoter, whereas the translation of the firefly luciferase (FLUC) is regulated by the 5*′* leader sequence of *TL1*-*binding transcription factor* (*TBF1*) (*5′LS_TBF1_*). Our previous study showed that translation of the reporter is normally inhibited by uORFs found in the *5′LS_TBF1_*.^14^ In response to pathogen challenge, there is a rapid but transient switch in translation from uORF to mORF through inhibition of uORF translation and activation of three purinerich “R-motifs” that serve as internal ribosome entry sites.^6,15–17^

**Figure 1.**
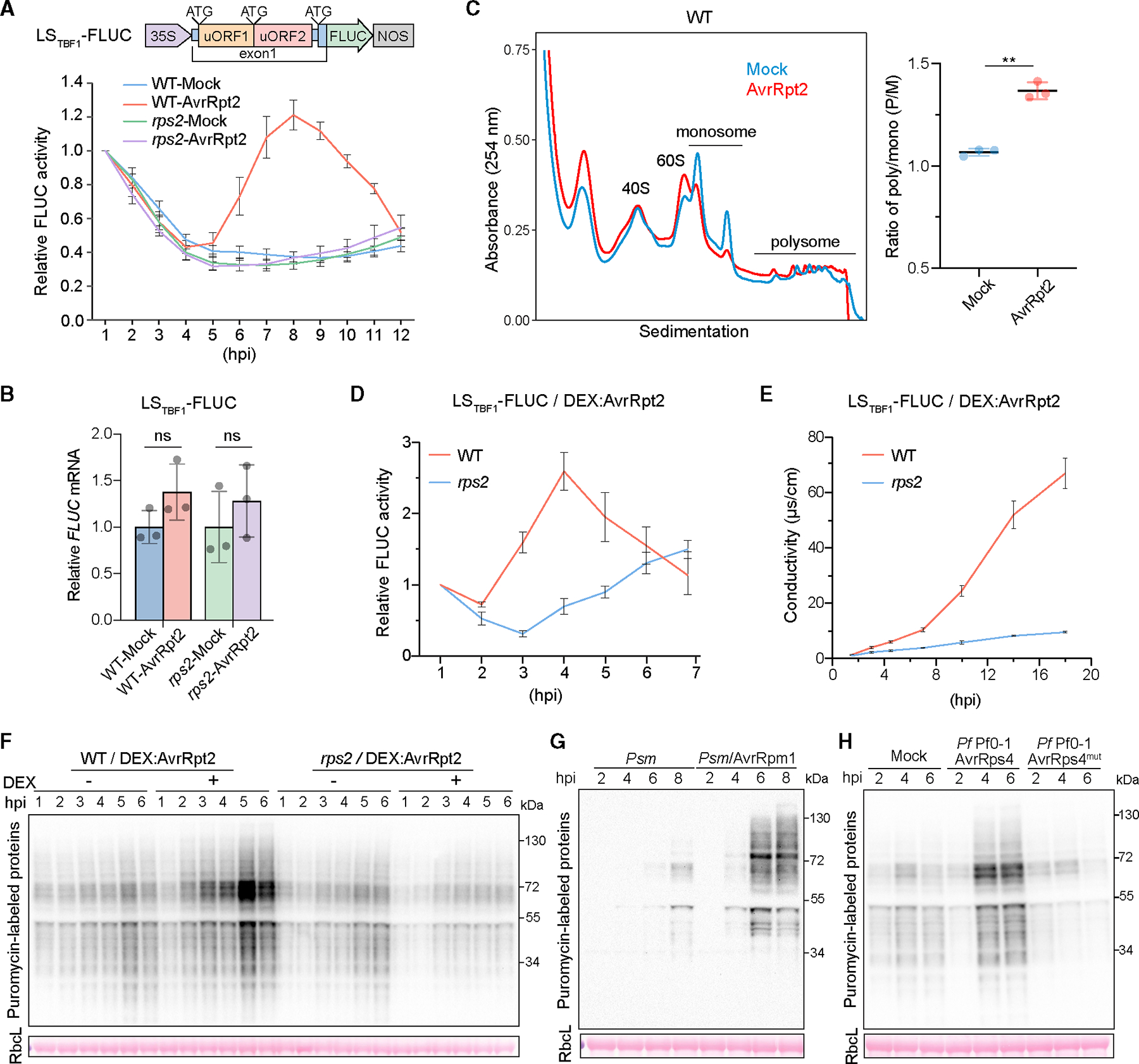
Global translational activity is elevated during ETI (A and B) Translational induction of the *LS*_*TBF1*_*-FLUC* reporter during *RPS2*-mediated ETI. WT or *rps2* plants with the reporter were inoculated with MgCl_2_ (Mock) or *Psm*/AvrRpt2 (AvrRpt2). FLUC activity (A) was normalized to 1 hpi for each genotype and presented as mean ± SEM (n = 12). *FLUC* mRNA level at 7 hpi (B) was normalized to Mock for each genotype and presented as mean ± SD (n = 3). Two-tailed Student’s t test; ns, not significant. (C) Polysome profiling of lysates from WT at 7 hpi with Mock or *Psm*/AvrRpt2. Polysome/monosome (P/M) ratios are presented as mean ± SD (n = 3). Two-tailed Student’s t test; **, p < 0.01. (D) Translational dynamics of the *LS*_*TBF1*_*-FLUC* reporter in WT or *rps2* plants carrying *DEX:AvrRpt2*. Data are presented as mean ± SEM (n = 6) after normalizing to 1 hpi of DEX for each genotype. (E) Conductivity assay measuring cell death upon DEX-induced expression of AvrRpt2. Data are presented as mean ± SEM (n = 3). (F–H) New protein synthesis during ETI was detected using the SUnSET assay. Leaf discs were treated with or without DEX (F). Plants in the Col-0 ecotype were infiltrated with *Psm* or *Psm*/AvrRpm1 (G) and in the Ws-2 ecotype were treated with *Pf* Pf0–1/AvrRps4 or *Pf* Pf0–1/AvrRps4^mut^ (H). RuBisCo large subunit (RbcL) was stained by Ponceau S as a loading control and was probably unlabeled in the SUnSET assay explaining the blank around 55 kDa in the immunoblot. See also Figure S1.

When plants carrying this translational reporter were inoculated with *Pseudomonas syringae* pv. *maculicola* (*Psm*) ES4326/AvrRpt2, a bacterial pathogen expressing AvrRpt2, the FLUC activity, not the *FLUC* mRNA, was induced in an RPS2-dependent manner with the peak time around 8 hours post inoculation (hpi) (Figures 1A and 1B). Based on the dynamics of the translational reporter, we examined the global translational activity using polysome profiling of Mock- and *Psm*/AvrRpt2-treated samples at 7 hpi, 1 h before the FLUC peak time. The results show that ETI decreases the abundance of monosome fractions while increasing the polysome fractions (Figure 1C). The increased ratio of polysome/monosome (P/M) in *Psm/*AvrRpt2-infected samples compared with Mock suggests that ETI enhances the global translational activity.

To further confirm this finding, which was missed in our previous study,^6^ we first demonstrated that the translational induction was due to the ETI response instead of signals from *Psm* by using the dexamethasone (DEX)-inducible AvrRpt2 plants (*DEX:AvrRpt2*).^18^ Similar to the *Psm/*AvrRpt2 challenge, DEX-mediated *in planta* expression of AvrRpt2 also induced the RPS2-dependent FLUC reporter activity (Figure 1D). Using these transgenic lines, we performed a surface sensing of translation (SUnSET) assay^19^ to evaluate the rate of protein synthesis during ETI. Puromycin, a structural analog of tyrosyl-tRNA that labels nascent peptides, was applied, and its incorporation rate was measured by immunoblotting from 1 to 6 hpi before massive cell death was detected (Figure 1E). Compared with Mock and *rps2*, more puromycin-labeled peptides were detected in the DEX-treated WT (Figure 1F), indicating that consistent with the polysome profiling, protein synthesis is indeed enhanced during ETI before cell death occurs.

Besides the RPS2 receptor, we also examined the translational changes during ETI mediated by other NLRs: Resistance to *P. syringae* pv. *maculicola* 1 (RPM1) (CNL) and RPS4, a Toll-like/interleukin-1 receptor (TIR)-NLR receptor (TNL), which detect bacterial effectors AvrRpm1 and AvrRps4, respectively.^20,21^ Similar to RPS2, activation by these NLRs elevated translational activities (Figures 1G and 1H), confirming that enhanced protein synthesis is a common phenomenon for ETI.

### CDC123 positively regulates ETI-mediated translation and resistance

To determine how translational activity is increased during ETI, we first tested the known stress-related translational regulators, eIF2α and GCN2. We found that while DEX-induced expression of AvrRpt2 alone was sufficient to trigger phosphorylation of eIF2α in an RPS2-dependent manner, *Psm* and *Psm*/AvrRpt2 had the same effect on eIF2α, indicating that eIF2α phosphorylation occurs not only during ETI but also during a successful infection (Figures S1A and S1B). However, this response was completely blocked in *gcn2* without a significant effect on the ETI-induced reporter or global translation (Figures S1C and S1D), indicating that GCN2 and its phosphorylation of eIF2α are not involved in ETI-mediated translational regulation.

**Figure 2.**
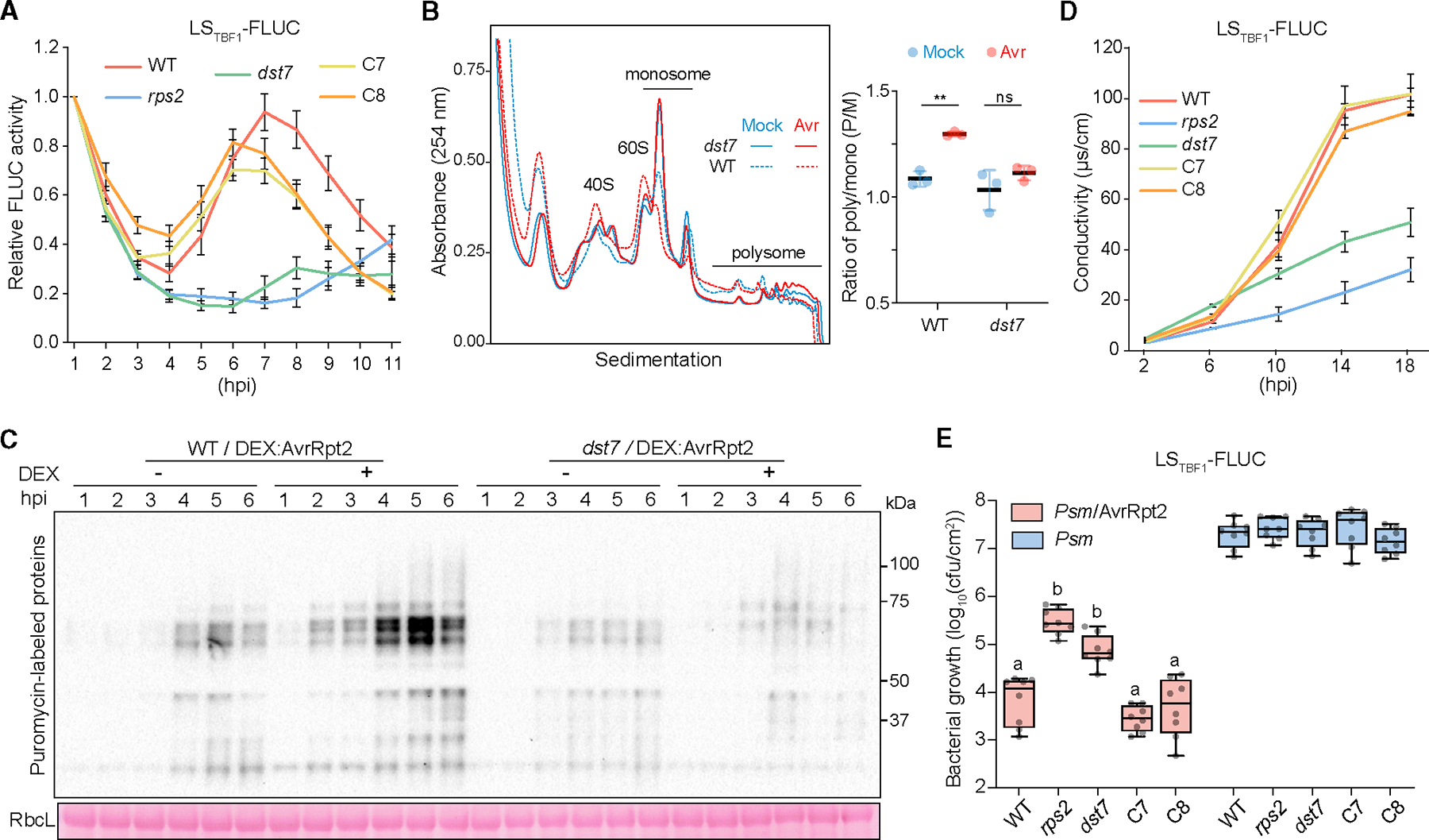
The *dst7* mutant is deficient in ETI-induced translation and defense (A) ETI-induced translational changes in *dst7* and the independent complementation lines, C7 and C8. Data are presented as mean ± SEM (n = 12) after normalizing to 1 hpi for each genotype. (B) Polysome profiling of WT and *dst7* at 7 hpi with Mock or *Psm*/AvrRpt2 (Avr). Polysome/monosome (P/M) ratios are presented as mean ± SD (n = 3). Two-tailed Student’s t test; **, p < 0.01; ns, not significant. (C) SUnSET analysis of WT and dst7 upon *DEX:AvrRpt2*-induced ETI. Ponceau S-stained RbcL was used as a loading control. (D) Conductivity assay measuring cell death induced by *Psm*/AvrRpt2 in *dst7* and the complementation lines. Data are presented as mean ± SEM (n = 4). (E) Bacterial growth in *dst7* and the complementation lines. Data are presented as a box-and-whisker plot (n = 8). Different letters indicate significant differences, one-way ANOVA, p < 0.05. See also Figure S2.

To uncover ETI translation regulators, we performed a genetic screen using the translational reporter and identified mutants with decreased suppression of reporter translation (*dst*). We selected the *dst7* mutant because it largely lost the responsiveness to translational induction by *Psm/*AvrRpt2 (Figure 2A), even though it had a higher-than-WT basal level of the reporter translation (Figures S2A–S2C). Backcrossing and whole-genome sequencing^22^ identified *dst7* as a recessive mutation in the *CDC123* gene (Figures S2D–S2F). It changes the aspartic acid (D) at residue 251, a conserved amino acid across species responsible for ATP binding,^23^ to an asparagine (N) (D251N) (Figures S2G and S2H). We then performed genetic complementation using two independent *35S:CDC123-YFP* lines (C7 and C8) in which the *dst7* morphology and reporter induction were restored to WT (Figures 2A, S2A–S2C, and S2I) without changing the reporter mRNA level (Figure S2J).

To determine the role of CDC123 in the ETI-mediated translational regulation, polysome profiling and the SUnSET assay were performed in *dst7* to evaluate its effect on the global translational activity. In contrast to WT, polysome profiling detected no significant shift between the monosome and polysome fractions in *dst7* after inoculation with *Psm*/AvrRpt2 (Figure 2B). This result was further confirmed in *dst7* carrying *DEX:AvrRpt2* (Figures 2C and S2K), indicating that CDC123 is a key regulator of ETI-associated translation.

Next, we examined *dst7* for ETI-associated PCD and pathogen resistance. We found that AvrRpt2-triggered PCD was largely compromised in *dst7* (Figures 2D and S2L), and this defect could be completely complemented by the *35S:CDC123-YFP* construct. The reduced resistance to *Psm*/AvrRpt2 in *dst7* was also rescued in the complementation lines (Figure 2E). These genetic data confirmed that the defect in CDC123 is the cause for the *dst7* phenotypes, and CDC123 plays a positive role in ETI.

### eIF2γ, a CDC123 interactor, is involved in ETI

**Figure 3.**
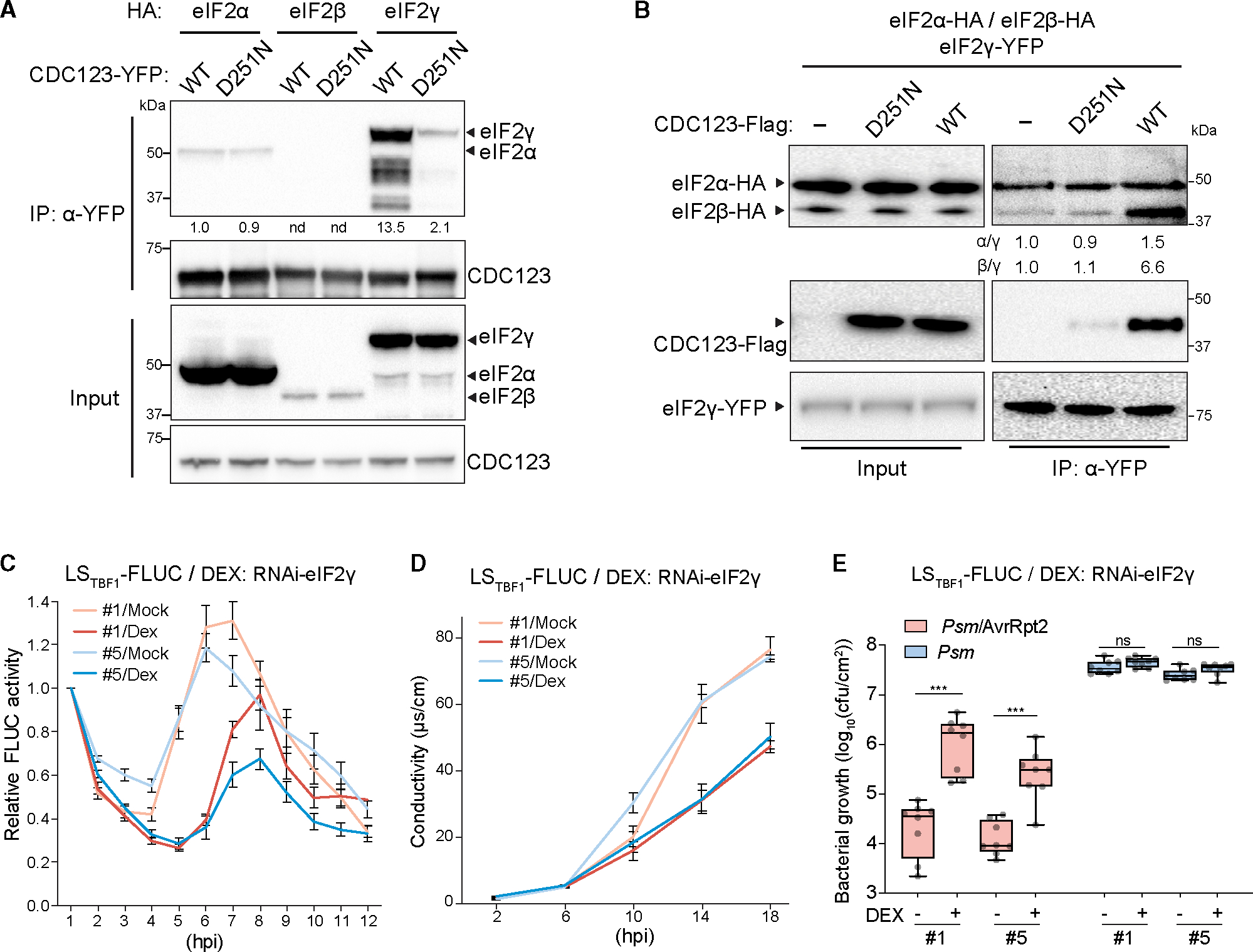
eIF2γ, an interactor of CDC123, is a positive regulator of ETI (A) Interactions between CDC123 and eIF2 subunits. Tagged proteins were transiently expressed in *N. benthamiana*. Numbers below the blot show relative band intensity normalized to the IP of CDC123-YFP. nd, not detected. (B) Effects of CDC123 on the eIF2 complex assembly. eIF2 subunits synthesized *in vitro* were incubated together with CDC123 (WT), CDC123-D251N (D251N), or control (−). Numbers below the blot show relative band intensity normalized to IP of eIF2γ-YFP. (C–E) ETI phenotypes of the inducible *eIF2γ*-silencing plants. FLUC activity (C) was normalized to 1 hpi of *Psm*/AvrRpt2 for each *DEX:RNAi-eIF2γ* line (#1 and #5, n = 12). Cell death rate (D) was assessed by conductivity measurement (n = 3). Data in (C) and (D) are presented as mean ± SEM. The bacterial population (E) is presented as a box-and-whisker plot (n = 8). Two-tailed Student’s t test; ***, p < 0.001; ns, not significant. See also Figure S3 and Table S1.

To investigate how CDC123 plays a role in ETI-mediated translational regulation, we performed immunoprecipitation-mass spectrometry (IP-MS) on the CDC123-YFP transgenic plants with or without the *Psm*/AvrRpt2 challenge (Figure S3A). Several translation initiation factors were found as potential CDC123 interactors (Figure S3B; Table S1), and eIF2γ was the most abundant among them, consistent with what is known for the yeast CDC123.^24^ We next examined the interaction between CDC123 and eIF2γ or other eIF2 subunits (α/β) *in planta* and detected an interaction with each subunit using the split luciferase complementation analysis (SLCA) in *Nicotiana benthamiana* (Figure S3C) and showed that the interactions occurred mainly in the cytoplasm based on the bimolecular fluorescence complementation (BiFC) assay (Figure S3D). However, in both the coIP and the yeast two-hybrid (Y2H) assays, CDC123 strongly interacted only with eIF2γ (Figures 3A and S3E), suggesting that in contrast to the direct interaction with eIF2γ, CDC123 interactions with eIF2α and eIF2β observed *in planta* might be indirect and/or transient, consistent with the yeast CDC123, which interacts with eIF2γ-eIF2α and eIF2γ-eIF2β intermediates through eIF2γ.^25^ Moreover, the D251N mutation only diminished the interaction with eIF2γ, but not with eIF2α (Figures 3A and S3F), similar to the yeast mutant of the conserved ATP-binding sequence (DIN).^23^

Based on these data, we hypothesized that the deficiency in ETI-induced translation and resistance in *dst7* is likely caused by the weakened interaction with eIF2γ, which reduces the available eIF2 complex. To test our hypothesis, we performed *in vitro* assembly assays using eIF2 subunits and the WT or mutated CDC123 that were individually expressed in a wheat germ translation system.^26^ In the subsequent coIP, we found that only the WT CDC123, not the D251N mutant, could enhance the interaction between eIF2γ and eIF2α or eIF2β, suggesting that the plant CDC123 plays a role in facilitating eIF2 assembly (Figure 3B).

If eIF2γ is a target of the CDC123 activity, it should also be involved in ETI-mediated translation and defense. Because of its essential function in translation, we utilized a DEX-inducible RNA interference transgene against *eIF2γ* (*DEX:RNAi-eIF2γ*) to transiently knock down its expression. To avoid the detrimental effect of inhibiting *eIF2γ* on seedling growth (Figure S3G), we applied DEX to mature plants for 3 days to moderately reduce *eIF2γ* RNA levels (Figure S3H). Under this condition, ETI-mediated reporter induction was delayed and weakened (Figure 3C). Moreover, similar to *dst7*, transient knockdown of *eIF2γ* was sufficient in blocking ETI-associated cell death and resistance, without perturbing basal resistance to *Psm* in the absence of AvrRpt2 (Figures 3D and 3E). These results demonstrate that consistent with eIF2γ being a substrate of CDC123, both proteins confer ETI-induced translational activity and resistance.

### ETI-elevated ATP concentration enhances the assembly of the eIF2 complex through CDC123

**Figure 4.**
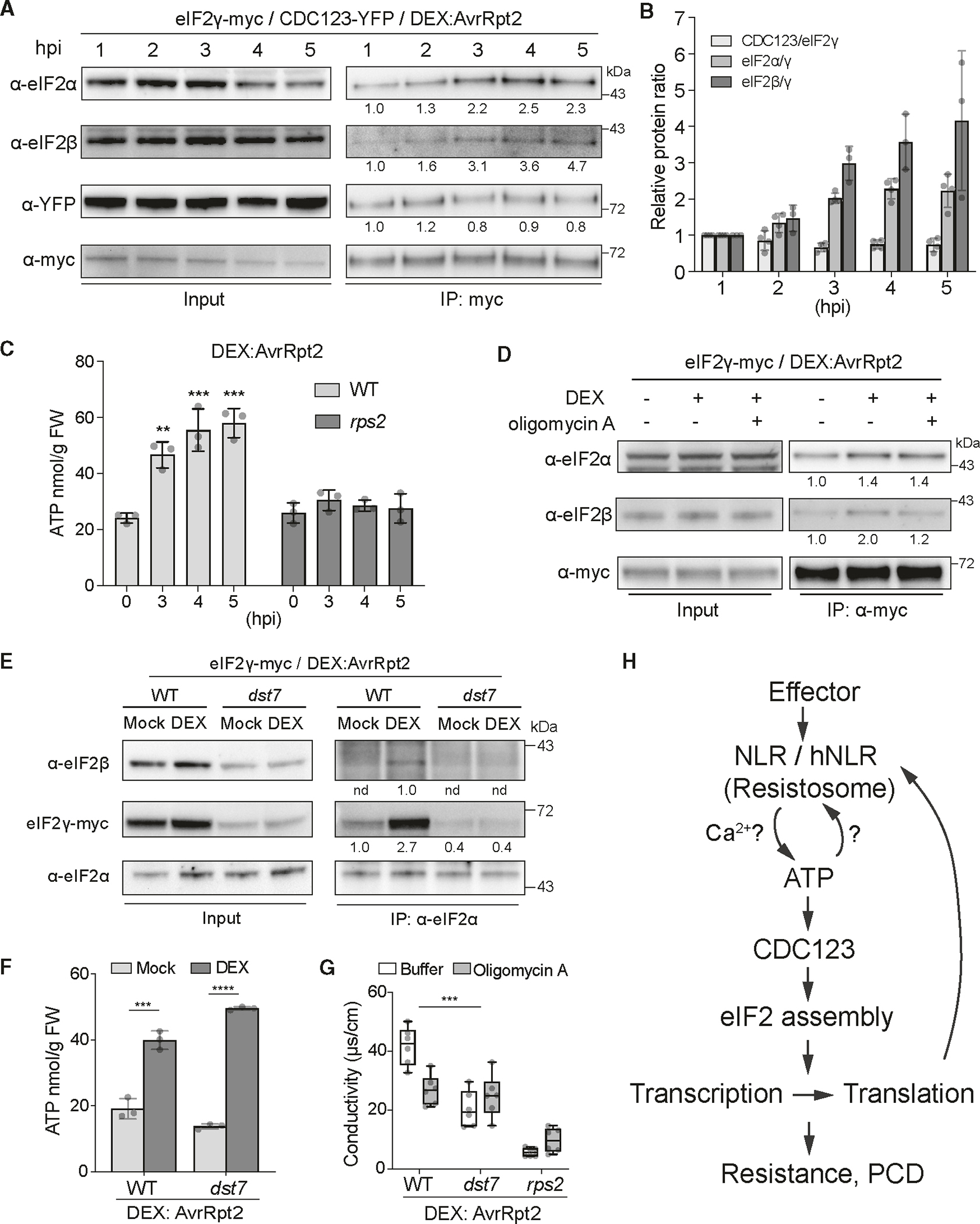
Elevated ATP level during ETI enhances CDC123-mediated eIF2 complex assembly to induce translation (A and B) Interaction dynamics of CDC123 and eIF2 subunits during *DEX*:*AvrRpt2*-induced ETI. Relative band intensity of the immunoblots (A) was normalized to IP of eIF2γ-myc (numbers below the blot) and their relative ratios (B) are presented as mean ± SD. (C) ATP level changes during ETI induced by *DEX:AvrRpt2* in WT or *rps2*. Data are presented as mean ± SD (n = 3). One-way ANOVA; **, p < 0.01; ***, p < 0.001. (D) The effect of oligomycin A on ETI-induced eIF2 assembly. Numbers below the blot show relative band intensity normalized to IP of eIF2γ-myc. (E) eIF2 complex assembly in WT or *dst7* plants upon ETI induction. Numbers below the blot show relative band intensity normalized to IP of eIF2α. nd, not detected. (F) ATP concentration in WT or *dst7* plants in response to *DEX:AvrRpt2* induction. Data are presented as mean ± SD (n = 3). Two-tailed Student’s t test; ***, p < 0.001; ****, p < 0.0001. (G) The effect of oligomycin A treatment on ETI cell death measured by the conductivity assay. Conductivity was measured at 16 hpi of DEX. Data are presented as a box-and-whisker plot (n = 6). Two-way ANOVA; ***, p < 0.0001. (H) Proposed model for the CDC123 function in regulating translation during ETI. See also Figure S4.

The genetic data on CDC123 and eIF2γ suggest that CDC123 might regulate ETI-associated translation by facilitating the assembly of the eIF2 complex. To detect the dynamics of the eIF2 complex during ETI, *35S:eIF2γ-myc* was introduced into the *DEX:AvrRpt2* background and further crossed with *35S:CDC123-YFP*. Time course of DEX induction was performed on the transgenic plants followed by coIP to examine the interactions between eIF2γ and eIF2α, eIF2β or CDC123. The results showed that the interaction between eIF2γ and eIF2α or eIF2β was enhanced from 3 to 5 hpi (Figures 4A and 4B), supporting our hypothesis that the eIF2 complex assembly is increased during ETI. Consistent with the role of CDC123 being a chaperone for the eIF2 complex assembly,^24^ its interaction with eIF2γ was slightly reduced during the ETI time course (Figures 4A and 4B).

Because CDC123 is an ATP-grasp protein and mutation of the ATP-binding site in *dst7* affects ETI-induced translation, we hypothesized that the CDC123 function in the eIF2 assembly might be regulated by ATP concentration changes during ETI. Time-course ATP measurement was conducted in WT and *rps2* plants carrying *DEX:AvrRpt2*. We found that ATP concentration was significantly increased 3–5 hpi in an RPS2-dependent manner (Figure 4C), correlating with the acceleration of eIF2 complex assembly (Figures 4A and 4B). This increase in ATP was also observed during ETI triggered by RPS4 in response to the WT effector AvrRps4 but not the mutant (Figure S4A), suggesting that ATP may be a common signal for CDC123 activation by both CNL and TNL classes of NLRs. To further validate this hypothesis, we applied ATPγS, a non-hydrolysable ATP that inhibits ATP-dependent enzyme activity, in an *in vitro* eIF2 assembly assay in the presence of CDC123. CoIP results showed that ATPγS attenuated the interaction between eIF2γ and eIF2α or eIF2β, supporting ATP being required for the eIF2 assembly (Figure S4B). Moreover, we applied an ATP synthesis inhibitor, oligomycin A, to block ETI-induced ATP concentration increase and tested the assembly of the eIF2 complex *in vivo*. CoIP results showed that oligomycin A treatment blocked the ETI-enhanced interaction between eIF2γ and eIF2β (Figure 4D). Overall, these results indicate that the increased ATP concentration during ETI induces the assembly of the eIF2 complex.

To demonstrate the dependence on CDC123 in ETI-mediated eIF2 assembly genetically, we crossed *35S:eIF2γ-myc* into *dst7* carrying *DEX:AvrRpt2* and performed coIPs to capture all three subunits of the eIF2 complex during ETI. Interestingly, we observed a dramatic reduction in the background level of the eIF2γ-myc protein in *dst7* (Figures 4E and S4C). Because this reduction in the protein was more than the decrease in the mRNA (Figures S4D and S4E), we hypothesize that CDC123 might affect eIF2γ protein stability. Using an anti-eIF2α antibody, we found that the DEX-induced increase in eIF2α interactions with eIF2β and eIF2γ detected in WT was diminished in *dst7* (Figure 4E). Similar results were observed when we used an anti-myc antibody against eIF2γ-myc in the coIP experiment (Figure S4C). These data confirmed that the ETI-induced eIF2 complex assembly is dependent on CDC123. Consistent with it being an ATP-insensitive mutant, *dst7* retains the ATP concentration increase upon ETI induction (Figure 4F). This result shows that in the absence of a functional CDC123, the ATP increase is not sufficient in facilitating eIF2 assembly. Indeed, we found that inhibition of ATP synthesis by oligomycin A treatment could compromise ETI-mediated translational induction and cell death in WT but not in *dst7* (Figures 4G and S4F).

## DISCUSSION

In this study, we identified CDC123, an ATP-grasp protein, as a key regulator of ETI-associated translational increase through enhanced eIF2 complex assembly (Figure 4H). Translational reprogramming during ETI has been suggested to be essential for mounting the local resistance, as well as for triggering systemic defense.^5,6,27,28^ Through characterization of the *cdc123* mutant, *dst7*, we provide a genetic proof that translational programming is specifically regulated and required for this immune response. A similar coordinated translational reprogramming may be required for pyroptosis in animals, a form of cell death that is also associated with the release of immune-stimulating signals.^29,30^

The ATP concentration increase triggered by RPS2 and RPS4 observed in our study (Figures 4C and S4A) and by RPM1 reported previously^31^ indicates that ATP is a common signal for both CNL- and TNL-mediated ETI. Though the basal ATP concentration may be sufficient for the initial activation of NLR, the subsequent increase in ATP levels may feedback amplify the response by activating additional helper NLRs (hNLRs) either directly or through enhancing their expression.^6,32^ Indeed, a recent mammalian study showed that upon immune induction, a sustained cytosol ATP concentration mediated by the activity of the mitochondrial electron transport chain is required for the NLR protein, NLRP3, to form the inflammasome.^33^ How the ATP concentration is elevated during ETI in plants remains unknown. One hypothesis is that the increase in ATP results from a lack of ATP consumption during ETI.^31^ This is unlikely to be correct because we have shown that translation, one of the most energy-consuming cellular processes, is dramatically enhanced during ETI. Alternatively, the ATP concentration increase may be driven by a mitochondrial Ca^2+^ uniporter^34,35^ in response to the cytoplasmic Ca^2+^ influx triggered by the resistosome. The elevated mitochondrial Ca^2+^ abundance may eventually enhance ATP synthesis to express and/or activate more NLRs to form an amplification loop (Figure 4H).

The deficiency in the eIF2 complex assembly due to the mutation in CDC123 explains not only the *dst7* mutant’s defect in ETI-mediated translation but also the deregulated *35S:5*′*LS_TBF1_-FLUC* reporter expression (Figures 2A and S2A–S2C), because the reduced eIF2 activity, while compromising NLR-mediated global translational induction, would allow the preinitiation complex to bypass the inhibitory uORF to translate the reporter. However, this study does not explain how the translation of the *35S:LS_TBF1_-FLUC* transcript containing two uORFs is normally induced in WT plants upon ETI induction, during which a dramatic increase in the reporter activity occurs independently of GCN2-mediated phosphorylation of eIF2α (Figure S1C). An alternative mechanism must be at play during ETI to allow the ribosome to bypass the uORF inhibition to translate the reporter and the endogenous defense transcripts containing uORFs.

Besides its function in translational regulation, *Arabidopsis* CDC123 was also identified as a regulator of DNA replication during cell cycle and pollen development.^36^ Because *dst7* identified in our study is viable, unlike the knockout mutant, whether it is deficient in DNA replication requires further investigation. Since our ETI experiments used fully expanded leaves, the ETI-deficient phenotype of *dst7* was unlikely to be impacted by a defect in cell cycle. In support of this, the BiFC images clearly indicate that CDC123 interacts with the eIF2 complex mainly outside the nucleus (Figure S3D). Moreover, the effect of CDC123 on translation is further supported by a similar ETI-deficient phenotype of the eIF2γ knockdown mutant (Figures 3C–3E).

Identification of CDC123 in the dynamic regulation of eIF2 assembly indicates that during ETI, translation is actively stimulated instead of being solely driven by mRNA levels. Transcriptional and translational reprogramming during ETI is likely to be coordinated by the increase in ATP concentration. Considering the conserved interactions between CDC123 and eIF2 subunits in yeast and humans,^25^ their role in translational regulation may be generally applied to eukaryotes in reprogramming the stress proteome.

## STAR★METHODS

### RESOURCE AVAILABILITY

#### Lead contact

Further information and requests for resources and reagents should be directed to and will be fulfilled by the lead contact, Xinnian Dong (xdong@duke.edu).

#### Materials availability

All unique plasmids and transgenic plants generated in this study are available from the lead contact upon completion of the Material Transfer Agreement.

#### Data and code availability

- This study generated a dataset provided in Table S1. This study does not report original codes.
- Any additional information required to reanalyze the data reported in this paper is available from the lead contact upon request.

### EXPERIMENTAL MODEL AND SUBJECT DETAILS

#### Plants

All *Arabidopsis thaliana* wild-type (WT), mutants, and transgenic plants used in this study are in the Columbia-0 (Col-0) ecotype background, except for the RPS4-related experiments in which the Wassilewskija-2 (Ws-2) ecotype was used. The *rps2* mutant,^12^ dexamethasone-induced AvrRpt2 (DEX:AvrRpt2) in WT and *rps2*,^39^ and *35S:5’LS_TBF1_-FLUC* in the WT and the *gcn2* mutant^16^ were described previously. Floral dip method^42^ was used to generate transgenic plants for constructs *35S:GFP* in WT, *35S:CDC123-YFP* in both the WT and the *dst7* backgrounds, *DEX:RNAi-eIF2*γ in the *35S:5’LS_TBF1_-FLUC* reporter line background, and *35S:eIF2*γ*-myc* in *DEX:AvrRpt2*. The *35S:5’LS_TBF1_-FLUC* reporter in *rps2*, *35S:CDC123-YFP/35S:eIF2*γ*-myc*/*DEX:AvrRpt2* in WT and *35S:eIF2*γ*-myc*/*DEX:AvrRpt2* in *dst7* were generated through genetic crosses. *Arabidopsis* and *N. benthamiana* plants were grown at 22 °C under a 12/12-hr light/dark cycle with 55% relative humidity for all the experiments except for plants used for transformation, which were grown at 22 °C under a 16/8-hr light/dark cycle.

#### Bacterial strains

*Pseudomonas syringae* pv. *maculicola* ES4326 (*Psm*) and *Psm* carrying the bacterial effector AvrRpt2 (*Psm/*AvrRpt2) or AvrRpm1 (*Psm*/AvrRpm1), and *Pseudomonas fluorescens* (*Pf*) Pf0–1 carrying AvrRps4 (*Pf* Pf0–1/AvrRps4) or the non-functional AvrRps4 KRVY-AAAA mutant (*Pf* Pf0–1/AvrRps4^mut^) were grown on King’s B plates at 30 °C for 2 days supplemented with appropriate anti-biotics.^21,38^ *Agrobacterium tumefaciens* strain GV3101 was grown in LB media at 30 °C.

### METHOD DETAILS

#### Plasmid construction

Plasmid information can be found in Table S2 where gene expression cassettes, bacterial antibiotic resistance and/or plant selection markers are specified. Briefly, CDC123 WT and D251N coding sequences were amplified from the intronless genomic regions of WT and the *dst7* mutant, respectively. eIF2α (At2g40290.2), eIF2β (At5g20920), and eIF2γ (At1g04170) were amplified from the ABRC clones U86660, U16002 and U16777, respectively, and inserted into modified pCAMBIA1300 vectors which contain the *35S* promoter and various tags through ligation-independent cloning.^43^ For *35S:CDC123-YFP* and *35S:eIF2*γ*-myc*, *hptII* in vectors was replaced with the *phosphinothricin* (*bar*) gene via *Xho*I as indicated in Table S2. For the yeast two-hybrid analysis, CDSs were inserted into the GAL4 AD or the BD vector via ligation-independent cloning. PCR products of eIF2γ fragment with different linker sequences were cloned into pRNAi-LIC as previously described.^44^ The hairpin cassette was cut by *Xho*I/*Spe*I and transferred into pBAV154.^45^ All the constructs were confirmed by Sanger sequencing before use.

#### Pathogen infection and conductivity assay

To measure bacterial growth in *Arabidopsis*, *Psm* and *Psm*/AvrRpt2 were resuspended in 10 mM MgCl_2_, diluted to OD_600nm_ = 0.002, infiltrated into fully expanded leaves and extracted 3 days post-infection (dpi) from 8 plants for each treatment. For the other assays, *Psm*, *Psm*/AvrRpt2, and *Psm*/AvrRpm1 were diluted to OD_600nm_ = 0.02, and *Pf* Pf0–1/AvrRps4 and *Pf* Pf0–1/AvrRps4^mut^ were infiltrated at OD_600nm_ = 0.2.

For the conductivity assay, 3–4 biological replicates with 6 discs each were collected at 1 hpi and washed with distilled water to measure ion leakage of dying cells using the ORION 3 STAR Portable Conductivity Meter (Thermo) at 22 °C starting at 2 hpi.

#### Dynamic FLUC activity recording

1 mM luciferin was sprayed on 3-week-old *Arabidopsis* plants 12 hr before treatment. For pathogen infection, leaves were infiltrated with *Psm*/AvrRpt2 at OD_600nm_ = 0.02 or 10 mM MgCl_2_ solution as Mock in the morning. For transgenic plants with *DEX:AvrRpt2*, 20 μM dexamethasone (DEX) or water (Mock) was sprayed on leaves. Starting at 1 hpi, FLUC activity was recorded every hour with each exposure for 20 min in the CCD camera-equipped box (Nightshade Company) under darkness, and measured as average grey intensity using Image J.

#### Polysome profiling

Polysome profiling was performed as previously described.^16^ Briefly, leaf tissues (0.4 g) were ground in liquid nitrogen and lysed in 1.6 ml of ice-cold polysome extraction buffer. After spinning down the debris, 1 ml of the lysate was loaded onto a 10.8 mL sucrose gradient (15–60%) and centrifuged at 35,000 rpm for 10 hr at 4 °C. Polysome profiles were generated using a fractionator and a 254 nm UV monitor.

#### SUnSET assay

SUnSET assay is modified from a previous protocol.^46^ Briefly, 4 leaf discs from 4-week-old plants were detached and floated in distilled water in 96-well plates. 50 μM puromycin was applied to samples for 40 min at each time point. Leaf discs were ground in liquid nitrogen, to which 80 μl of 2x SDS-sample buffer was subsequently added. After vigorous mixing, lysates were boiled at 95 °C for 10 min. Puromycin-labeled proteins were detected by western blot probed with an anti-puromycin antibody (Sigma).

#### Genetic screen and identification of the causal mutation

~2.5 g of the *35S:5’LS_TBF1_-FLUC* seeds were mutagenized using ethyl methanesulfonate (EMS). Briefly, seeds were soaked in 40 ml of 100 mM potassium phosphate buffer (pH 7.4) at 4 °C overnight followed by 0.4% (v/v) EMS treatment in fresh 40 ml of 100 mM potassium phosphate buffer for 8 hr at room temperature on a rotating platform. Seeds were then washed 20 times with water and dried on filter paper before ~ 7500 of them were sowed in 40 pots (20 cm* 20 cm). M2 seeds were collected in 40 pools, and 800 seeds from each pool were sowed on 1/2 MS plates (containing 1% sucrose and 0.8 % agar). After growth in a chamber for 12 days, seedlings were placed in darkness for 12 hr and then sprayed with 1 mM luciferin. After the solution was air-dried, FLUC in seedlings was imaged using a CCD camera-equipped box. The *dst* mutants were identified for their elevated *35S:5’LS_TBF1_-FLUC* expression. The FLUC and defense phenotypes were then confirmed in the M3 plants. To exclude mutants of *35S:5’LS_TBF1_-FLUC*, a sequencing analysis of the reporter was performed. For the *dst7* mutant, the segregation of FLUC and growth phenotypes was confirmed in the bcF2 plants. From this population, leaves of 96 individuals with the *dst7* phenotype were pooled in parallel with the pool of 50 plants with WT phenotype for genomic DNA extraction using DNeasy Plant Mini Kit (Qiagen) and sent to the Duke Center for Genomic and Computational Biology for library construction and whole-genome sequencing using the Illumina Hi-Seq2000 platform. Compared to the WT/*35S:5’LS_TBF1_-FLUC* reference genome, SNPs were called from the *dst7* mutant samples using two previously described methods, NGM and SNPtrack.^22,41^ The same mutation sites were identified using these two methods.

#### Quantitative real-time PCR

Total RNA was isolated from ~100 mg of leaf tissue using TRIzol (Ambion). From the resulting RNA samples, DNA contamination was removed using DNase I (Ambion) and reverse transcription was performed using SuperScript^®^ III Reverse Transcriptase (Invitrogen) with an oligo(dT) primer. Real-time PCR was done using FastStart Universal SYBR Green Master (Roche) with primers listed in Table S2. *Ubiquitin 5* (*UBQ5*) was used as the internal control.

#### LC-MS/MS and data analysis

3-week-old *35S:GFP* and *35S:CDC123-YFP* transgenic *Arabidopsis* plants were infiltrated in the morning with *Psm*/AvrRpt2 at OD_600nm_ = 0.02 or 10 mM MgCl_2_ solution as Mock 1 hr before being placed in darkness for 7 hr when the translational induction reaches the peak based on the FLUC data collected from the LS*_TBF1_*-FLUC/WT plants. 2 g of leaf tissue was collected, and protein was extracted in the immunoprecipitation (IP) buffer [50 mM Tris, pH 7.5, 150 mM NaCl, 0.1% Triton X-100, 0.2% Nonidet P-40, plant protease inhibitor cocktail (Roche), and 40 μM MG115]. IP was performed using GFP-Trap beads overnight at 4 °C. Beads were washed three times with the wash buffer (50 mM Tris, pH 7.5 and 150 mM NaCl) and twice with the 1xPBS buffer (pH 7.4). On-bead trypsin digestion, peptide lyophilization and LC-MS/MS were performed by the Duke Proteomics Core Facility. The data were processed using Proteome Discoverer, and a database search was performed using the mascot server v2.4 against the *Arabidopsis* proteome from NCBI (RefSeq). The data were then curated and analyzed using the Scaffold v4 software (Proteome Software, Inc.). Potential interactors of CDC123 were identified based on the criteria that the peptides were only detected in the CDC123 sample (at least 4 spectrum counts) or enriched by 2 fold (Table S1). The ratio of CDC123 interactors upon ETI was calculated by comparing the spectrum counts after normalization to the total peptides of CDC123 in Mock and AvrRpt2 treatments, respectively.

#### Transient gene expression in *N. benthamiana*

*Agrobacterium* GV3101 was used for transient gene expression in *N. benthamiana* as described previously.^44^ Briefly, bacteria were cultured overnight in 5 ml liquid Luria-Bertani (LB) media at 30 °C, spun down, resuspended in the infiltration buffer (10 mM MgCl_2_, 10 mM MES pH = 5.7, 200 μM acetosyringone) and mixed at OD_600nm_ = 0.2 each for infiltration.

#### Co-immunoprecipitation and protein analysis

*Agrobacteria* containing *35S:CDC123-YFP or 35S:CDC123-D251N-YFP* were mixed with those expressing *35S:eIF2α-HA*, *35S:eIF2β-HA* or *35S:eIF2*γ*-HA*, and inoculated into *N. benthamiana* for transient gene expression for 24 hr. Leaf tissue was ground in liquid nitrogen and lysed in the IP buffer for 0.5 hr. The lysate was incubated with 20 μl GFP-Trap beads (Chromotek) for 4 hr at 4 °C. For transgenic *Arabidopsis* with *DEX:AvrRpt2* induction, samples of the time-course experiment were collected every hour from 1 hpi, and other samples with treatments were collected at 4 hpi. Leaf tissues with or without treatment were lysed in the IP buffer, and the lysate was incubated with 20 μl anti-myc magnetic beads (ThermoFisher) to IP eIF2γ-myc. For IP with eIF2α antibody, 1.5 mL lysate was incubated with 10 μg antibody overnight, and further mixed with 50 μl protein A agarose beads. Extra lysate mixed with 4x SDS-sample buffer was boiled and saved as input. After incubation, beads were washed with the IP buffer for 3 times and boiled in 2x SDS-sample buffer. For total protein extraction, 4 leaf discs were sampled and ground in liquid nitrogen. Samples mixed with 80 μl 2x SDS-sample buffer were boiled for 10 min before SDS-PAGE gel analysis. The band intensity in blots was calculated using ImageJ.

#### Bimolecular fluorescence complementation assay

*Agrobacteria* containing *35S:CDC123-nYFP* were mixed with those carrying *35S:eIF2α-cYFP*, *35S:eIF2β-cYFP* or *35S:eIF2γ-cYFP* and inoculated into *N. benthamiana* for transient gene expression for 40 hr. YFP fluorescence in leaf discs was observed using an inverted Zeiss LSM 510 laser scanning microscope.

#### Split luciferase complementation assay

Split luciferase complementation assay (SLCA) was performed as described previously.^47^ *Agrobacteria* containing *35S:CDC123-nLUC* were co-infiltrated with those carrying *35S:eIF2α-cLUC*, *35S:eIF2β-cLUC* or *35S:eIF2γ-cLUC* into *N. benthamiana* for transient gene expression for 40 hr. Leaves were detached and sprayed with 1 mM luciferin and FLUC imaging was done using the ChemiDoc^™^ XRS+ system (Bio-Rad).

#### Yeast two-hybrid analysis

Matchmaker^™^ GAL4 yeast two-hybrid system was used by following the manufacturer’s instructions (PT3247–1). Co-transformation was used for pairwise interaction tests.

#### *In vitro* eIF2 complex assembly

Proteins used for the *in vitro* assembly assay were synthesized using a wheat germ translation system according to the manufacturer’s instruction (Sigma). For the assembly assay, proteins were mixed in equal proportions and incubated at room temperature for 1 hr. For the ATP dependency experiment, 10 mM nonhydrolyzable ATP, ATPγS, was added to the mixture. Protein mixtures were further diluted in the IP buffer and immunoprecipitated with GFP-Trap or anti-myc beads at 4 °C.

#### ATP measurement

*Arabidopsis* leaves (~0.1 g) were weighed (fresh weight, FW), ground in liquid nitrogen, and extracted in 0.2 ml of 5% trichloroacetic acid. Lysates were diluted 50 times with distilled H_2_O and subjected to measurement using ATP Bioluminescent Assay Kit (Sigma).

#### Chemical treatment

For induction of *DEX:AvrRpt2* or *DEX:RNAi-eIF2γ*, 20 μM dexamethasone (DEX) or water (Mock) was applied. For the SUnSET assay, 20 μM DEX was added in wells with leaf discs. After 2.5 hr of 20 μM DEX treatment, oligomycin A (20 μM) was applied to leaf discs for 1.5 hr. For co-IP and conductivity experiments, plants were first sprayed with 20 μM DEX, 2.5 hr later, infiltrated with 5 μM oligomycin A diluted in buffer (0.001% TritonX-100) or buffer alone, and then sampled at 4 hpi.

### QUANTIFICATION AND STATISTICAL ANALYSIS

Normal distribution was determined using the Shapiro-Wilk test. Two-tailed Student’s t-test and ANOVA were used for two and multiple comparisons (post-hoc analysis with the Tukey test), respectively. Asterisks in all graphs indicate statistical significance (*, p < 0.05; **, p < 0.01; ***, p < 0.001; ****, p < 0.0001, ns, not significant). For one-way ANOVA with multiple samples comparison (Figure 2E), different lowercase letters indicate significant differences (p < 0.05). Statistical tests were performed using GraphPad Prism 8. Unless specifically stated, sample size n means biological replication, each dot in graphs represents a biological replicate, and experiments have been performed at least three times. Other information about statistical parameters can be found in figure legends.

## Supplementary Material

Supplemental figures

Supplemental table 1

Supplemental table 2

## Figures and Tables

**KEY RESOURCES TABLE:** 

REAGENT or RESOURCE	SOURCE	IDENTIFIER

Antibodies		

Mouse monoclonal anti-GFP (JL8)	Clontech	Cat. #632381; RRID: AB_2313808
Mouse monoclonal anti-c-Myc (9E10)	Santa Cruz	Cat. #sc-40; RRID: AB_627268
Mouse monoclonal anti-HA (6E2), HRP Conjugated	CST	Cat: #2999; RRID: AB_1264166
Mouse monoclonal anti-FLAG	Sigma-Aldrich	Cat. #F1804; RRID: AB_262044
Mouse monoclonal anti-Luciferase	Sigma-Aldrich	Cat. #L2164; RRID: AB_439707
Mouse monoclonal anti-Puromycin (12D10)	Sigma-Aldrich	Cat. #MABE343; RRID: AB_2566826
Rabbit polyclonal anti-EIF2α	Sigma-Aldrich	Cat. #SAB4500729; RRID: AB_10745021
Rabbit monoclonal phospho-eIF2α (Ser51)	Abcam	Cat. #ab32157; RRID: AB_732117
Rabbit antiserum anti-eIF2β	Dennis and Browning^37^	N/A

Bacterial and virus strains		

Agrobacterium *tumefaciens* strain GV3101	N/A	N/A
Pseudomonas *syringae* pv. *maculicola* ES4326/AvrRpt2	Reuber and Ausubel^38^	N/A
Pseudomonas *syringae* pv. *maculicola* ES4326/AvrRpm1	Reuber and Ausubel^38^	N/A
Pseudomonas *fluorescens* Pf0-1/AvrRps4	Sohn et al.^21^	N/A
Pseudomonas *fluorescens* Pf0-1/AvrRps4^mut^ (KRVY-AAAA)	Sohn et al.^21^	N/A

Chemicals, peptides, and recombinant proteins		

Dexamethasone	Sigma-Aldrich	Cat. #D1756-25MG
D-Luciferin, Potassium Salt	Goldbio	Cat. #LUCK-100
Oligomycin A	Sigma-Aldrich	Cat. #75351
ATPγS	Sigma-Aldrich	Cat. #A1388
GFP-Trap Agarose	ChromoTek	Cat. #gta-20
Anti-c-Myc Magnetic Beads	Thermo Scientific	Cat. #88843

Critical commercial assays		

DNeasy Plant Mini Kit	Qiagen	Cat. #69104
ATP Bioluminescent Assay Kit	Sigma-Aldrich	Cat. #FLAA
Next Generation Cell Free Protein Expression Kit (Wheat Germ)	Sigma-Aldrich	Cat. #CFPS700
FastStart Universal SYBR Green Master Kit	Roche	Cat. #04913850001

Experimental models: Organisms/strains		

*Arabidopsis:* Col-0	N/A	N/A
*Arabidopsis:* Ws-2	N/A	N/A
*Arabidopsis: 35S:5’LS_TBF1_-FLUC*	Xu etal.^16^	N/A
*Arabidopsis: rps2/35S:5’LS_TBF1_-FLUC*	This paper	N/A
*Arabidopsis: gcn2/35S:5’LS_TBF1_-FLUC*	Xu etal.^16^	N/A
*Arabidopsis: DEX:AvrRpt2*	Axtell et al.^18^	N/A
*Arabidopsis: rps2/DEX:AvrRpt2*	Gu et al.^39^	N/A
*Arabidopsis: 35S:5’LS_TBF1_-FLUC/DEX:AvrRpt2*	This paper	N/A
*Arabidopsis: rps2/35S:5’LS_TBF1_-FLUC/DEX:AvrRpt2*	This paper	N/A
*Arabidopsis: dst7*	This paper	N/A
*Arabidopsis: dst7/35S:CDC123-YFP*	This paper	N/A
*Arabidopsis: dst7/DEX:AvrRpt2*	This paper	N/A
*Arabidopsis: 35S:CDC123-YFP*	This paper	N/A
*Arabidopsis: 35S:5’LS_TBF1_-FLUC/DEX:RNAi-eIF2r*	This paper	N/A
*Arabidopsis: 35S:eIF2r-myc/DEX:AvrRpt2*	This paper	N/A
*Arabidopsis: 35S:eIF2r-myc/35S:CDC123-YFP/DEX:AvrRpt2*	This paper	N/A
*Arabidopsis: dst7/35S:eIF2r-myc/DEX:AvrRpt2*	This paper	N/A

Oligonucleotides		

Primers see Table S2		

Recombinant DNA		

35S:5’S_TBF1_-FLUC	Xu etal.^16^	N/A
35S:CDC123(WT/D251N)-YFP	This paper	N/A
35S:CDC123-nYFP/nLUC	This paper	N/A
35S:eIF2α-HA/cYFP/cLUC	This paper	N/A
35S:eIF2β-HA/cYFP/cLUC	This paper	N/A
35S:eIF2γ-myc/HA/cYFP/cLUC	This paper	N/A
AD-CDC123(WT/D251 N)	This paper	N/A
BD-eIF2α/eIF2β/eIF2γ	This paper	N/A
DEX:RNAi-eIF2γ	This paper	N/A

Software and algorithms		

ImageJ (FIJI)	Schindelin et al.^40^	https://imagej.nih.gov/ij/
Prism 8	GraphPad	https://www.graphpad.com/
Scaffold v4	Proteome Software	N/A
NGM	Austin et al.^22^	http://bar.utoronto.ca/NGM/
SNPtrack	Leshchiner et al.^41^	http://genetics.bwh.harvard.edu/snptrack/

## Highlights

- Effector-triggered immunity (ETI) elevates global translational activity
- A genetic screen identifies CDC123 as a positive regulator of translation during ETI
- Translation initiation factor eIF2γ interacts with CDC123 and positively regulates ETI
- ATP increase during ETI enhances CDC123-mediated eIF2 complex assembly and translation
