# Supplementary material for: Global translational induction during NLR-mediated immunity in plants is dynamically regulated by CDC123, an ATP-sensitive protein
Source: Cell Host Microbe. Author manuscript; Available in PMC 2024 Feb 27. (PMC10898606; doi:10.1016/j.chom.2023.01.014)
Supplement: Supplemental figures [file NIHMS1966742-supplement-Supplemental_figures.pdf]

SUPPLEMENTAL FIGURES

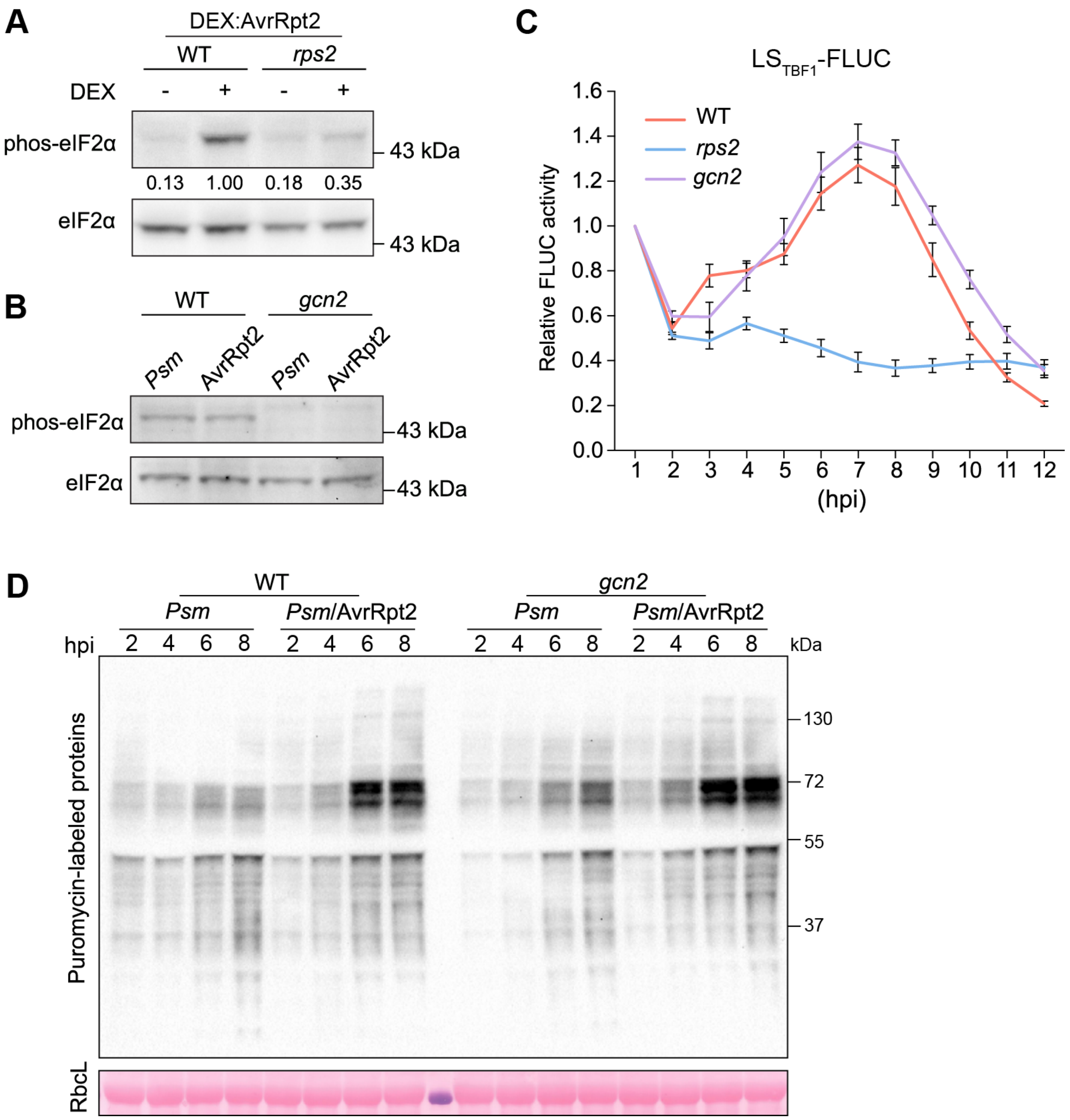

Figure S1. GCN2-mediated eIF2α phosphorylation is not required for ETI-associated translation. Related to Figure 1.

(A) eIF2α phosphorylation in WT or the *rps2* mutant during *DEX:AvrRpt2*-induced ETI. Plants were sprayed with 20 μM DEX and samples were tested at 4 hpi. The numbers

indicate ratios of phosphorylated eIF2α to total eIF2α and were normalized to the DEX-treated WT sample.

(B) eIF2α phosphorylation in WT or the *gcn2* mutant during *Psm* or *Psm*/AvrRpt2 (AvrRpt2) challenge. Plants were infiltrated with bacteria, and samples were tested at 7 hpi.

(C) The translational dynamics of the LS_{TBF1}-*FLUC* reporter in WT, *rps2* or *gcn2* plants in response to *Psm*/AvrRpt2 inoculation. Data are presented as mean ± SEM (n = 12) after normalizing to 1 hpi for each genotype.

(D) SUnSET analysis of WT and *gcn2* upon *Psm* or *Psm*/AvrRpt2 inoculation. Ponceau S-stained RbcL was used as a loading control.

A

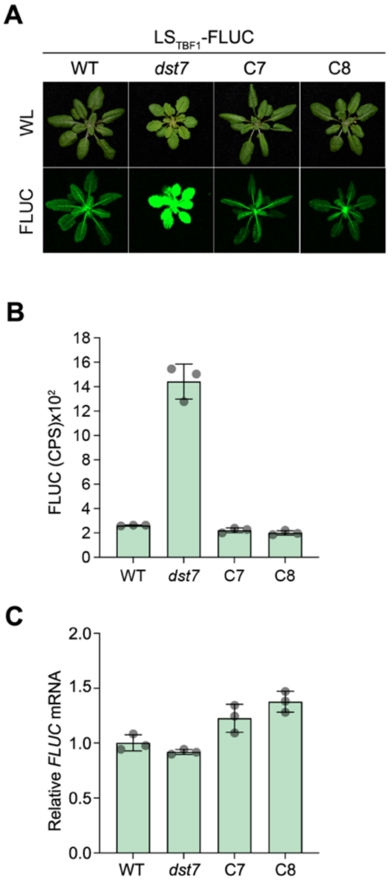

D

Generation	Phenotype: Growth	Phenotype: FLUC	Genotyping method	Genotype: LPK1 (AA)	Genotype: CDC123 (BB)
LS_{TBF1}-FLUC	Normal	Peak	PCR-seq	AA	BB
M2 (*dst7*)	Small	No	PCR-seq	aa	bb
bcF2 (133/538, P=0.881)	Small	No	whole-genome seq	1A/43a (reads)	0B/70b (reads)
bc2F2 (9/35, P=0.922)	Small	No	*Eae*I/*Apo*I/PCR-seq	Aa (9/9)	bb (9/9)
bc3F2 (6/25, P=0.908)	Small	No	*Eae*I/*Apo*I/PCR-seq	AA (6/6)	bb (6/6)

· ***P*: two-tailed *P* value, χ^2 test**

· **Growth and FLUC phenotypes are linked**

E

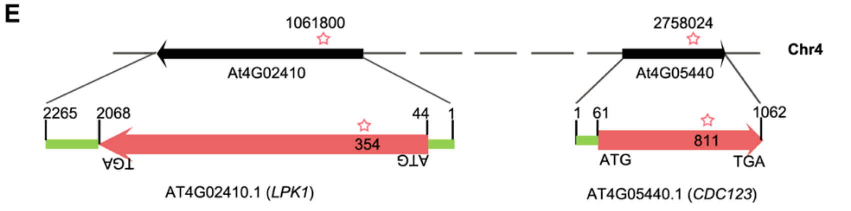

F

EaeI/ApoI/PCR-seq

Primers	Length (bp)	LPK1-EaeI (bp): AA	LPK1-EaeI (bp): Aa	LPK1-EaeI (bp): aa	CDC123-ApoI (bp): BB	CDC123-ApoI (bp): Bb	CDC123-ApoI (bp): bb
**G715/G819**	**642**	**310+342**	**642+310+342**	**642**			
**G868/G869**	**316**				**316**	**316+252+64**	**252+64**

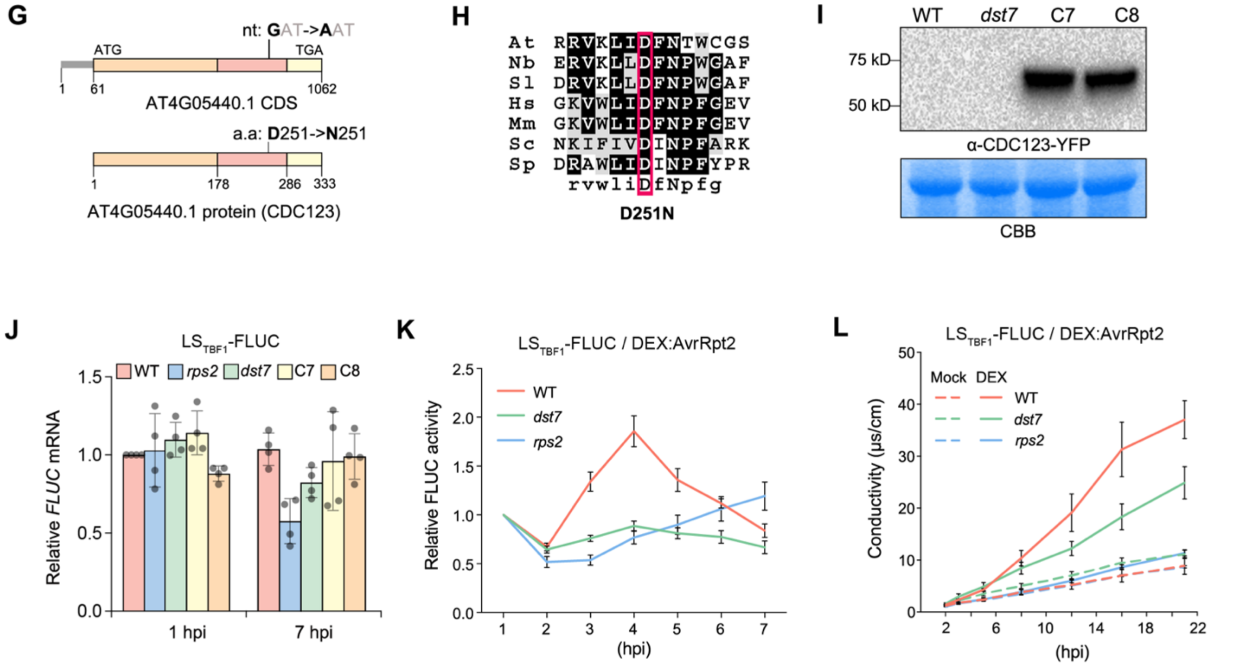

Figure S2. Identification of CDC123 as a translational regulator of ETI through analyses of the *dst7* mutant. Related to Figure 2.

(A-C) Phenotypes of *dst7* and complementation plants with representative pictures (A) and quantification of their basal FLUC activities (B) and *FLUC* mRNA levels (C). C7 and C8, two independent *dst7* lines complemented with *35S:CDC123-YFP*; WL, white light. FLUC activity was measured as photon counts per second (CPS) from the lysates. Data are presented as mean ± SEM (n = 3) of CPS for (B) and mean ± SD (n = 3) of the *FLUC* mRNA.

(D) Identification of the causal gene for the *dst7* mutant phenotype. Plant growth and translational induction of the LS_{TBF1}-*FLUC* reporter in the parental lines are defined as "normal" and "peak", respectively, while the *dst7* mutant plants are small with no FLUC induction. The *dst7* mutant was backcrossed (bc) with the parental line, and whole-genome sequencing was performed using pooled bcF2 with a small size, which led to the identification of *LPK1* and *CDC123* (aa and bb) as potential candidate genes. *LPK1* was ruled out as the candidate based on the segregation of the gene from the *dst7* phenotypes observed in the second and third backcrosses (bc2F2 and bc3F2).

(E) Two homozygous mutations in the *dst7* mutant. The black arrow, red arrow, green rectangle, and red star represent genomic regions, CDS regions, UTRs, and mutation sites, respectively.

(F) Genotyping of the mutation sites in *LPK1* and *CDC123*. Mutation in *LPK1* causes the loss of the *Eae*I site, while mutation in *CDC123* gains an *Apo*I site. Primer sequences are in Table S2.

(G) Gene and protein structures of CDC123. The *dst7* mutant has a G811A mutation in *CDC123* (AT4G05440.1), which causes the D251N change in the protein.

(H) Sequence alignment of CDC123 homologs in different species. At (*Arabidopsis thaliana*; AT4G05440.1), Nb (*Nicotiana benthamiana*; Niben101Scf39514g00002.1), Sl (*Solanum lycopersicum*; Solyc01g107630.2.1), Hs (*Homo sapiens*; NP_006014), Mm (*Mus musculus*; NP_598598.1), Sc (*Saccharomyces cerevisiae*; Q05791), and Sp (*Schizosaccharomyces pombe*; Q9P7N5). The conserved D251 is highlighted with the red rectangle.

(I) The CDC123-YFP protein level in the complementation lines with Coomassie brilliant blue (CBB) staining as a loading control.

(J) The *FLUC* mRNA levels at 1 and 7 hpi with *Psm*/AvrRpt2. Data are presented as mean ± SD (n = 3) after normalizing to that at 1 hpi.

(K) Translational level of the *LS*$_{TBF1}$*-FLUC* reporter upon DEX induction. The FLUC intensity was normalized to that at 1 hpi and presented as mean ± SEM (n = 6).

(L) Cell death induced by *DEX:AvrRpt2* measured by the conductivity assay. Data are presented as mean ± SD (n = 3).

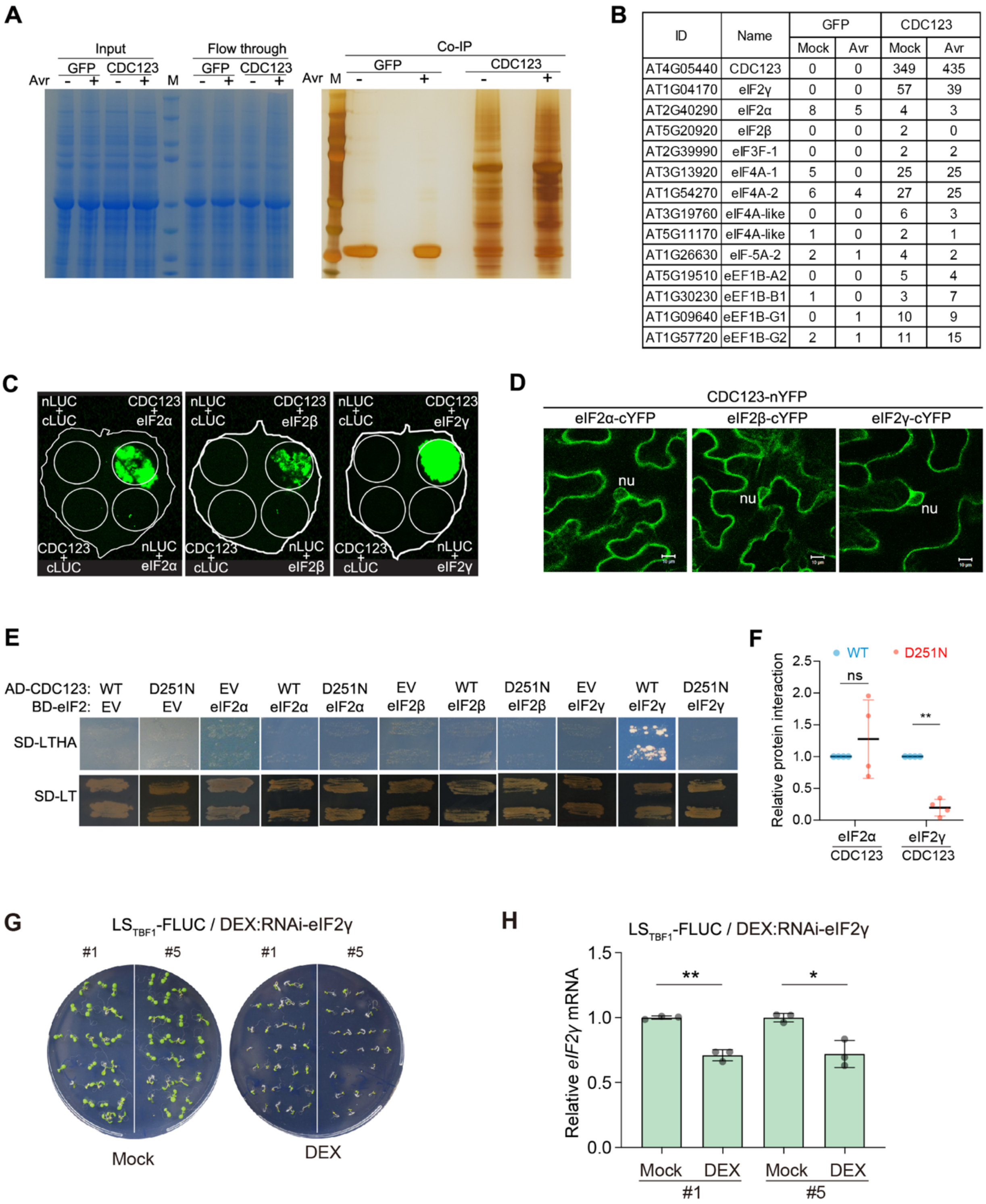

ID	Name	GFP		CDC123	
		Mock	Avr	Mock	Avr
AT4G05440	CDC123	0	0	349	435
AT1G04170	eIF2γ	0	0	57	39
AT2G40290	eIF2α	8	5	4	3
AT5G20920	eIF2β	0	0	2	0
AT2G39990	eIF3F-1	0	0	2	2
AT3G13920	eIF4A-1	5	0	25	25
AT1G54270	eIF4A-2	6	4	27	25
AT3G19760	eIF4A-like	0	0	6	3
AT5G11170	eIF4A-like	1	0	2	1
AT1G26630	eIF-5A-2	2	1	4	2
AT5G19510	eEF1B-A2	0	0	5	4
AT1G30230	eEF1B-B1	1	0	3	7
AT1G09640	eEF1B-G1	0	1	10	9
AT1G57720	eEF1B-G2	2	1	11	15

Figure S3. CDC123 interacts with eukaryotic translation initiation factor eIF2γ. Related to Figure 3.

(A and B) Identification of translational regulators that interact with CDC123 using Mass-spectrometry (MS). The *35S:GFP* (GFP) and the *35S:CDC123-YFP* (CDC123) transgenic plants were treated with 10 mM $MgCl_2$ or *Psm*/AvrRpt2 (Avr) to generate input and co-IP (A) samples for MS. Total protein and flow through were evaluated by Coomassie brilliant blue staining. Silver staining was performed to detect the abundance of interactors. M, protein marker. This experiment was performed once with the total spectrometry counts of translation-related factors shown in (B).

(C) Interactions between CDC123 and eIF2 subunits were detected by the split luciferase complementation assay (SLCA). nLUC, N terminal half of FLUC; cLUC, C terminal half of FLUC.

(D) Interactions between CDC123 and eIF2 subunits were detected using the bimolecular fluorescence complementation (BiFC) assay. nYFP, N terminal half of YFP; cYFP, C terminal half of YFP. nu: nucleus. Scale bar, 10 μm.

(E) Yeast two-hybrid detection of interactions between WT or mutated CDC123 and eIF2 subunits. Two individual colonies were shown for each co-transformation. Interaction is indicated by growth on the medium lacking Histidine/Adenine (SD-LTHA). EV, empty vector; AD, GAL4 activation domain; BD, GAL4 DNA binding domain; WT, wild-type CDC123; D251N, CDC123 mutant protein identified in *dst7*.

(F) Co-IP of WT CDC123 or the D251N mutant with eIF2 subunits. The ratio of indicated proteins was normalized to WT CDC123 in each replicate (n = 4). Two-tailed Student's *t*-test.

(G) Effects of silencing *eIF2γ* on seedling growth. The *DEX:RNAi-eIF2γ* construct was transformed into plants carrying the LS_{TBF1}*-FLUC* reporter. Two independent transgenic lines (#1 and #5) were grown on plates with sterile water (Mock) or 20 μM DEX for 6 days.

(H) Endogenous *eIF2γ* mRNA levels 3 days after DEX induction of *RNAi-eIF2γ* in mature leaves. Data are presented as mean ± SD (n = 3). Two-tailed Student's *t*-test.

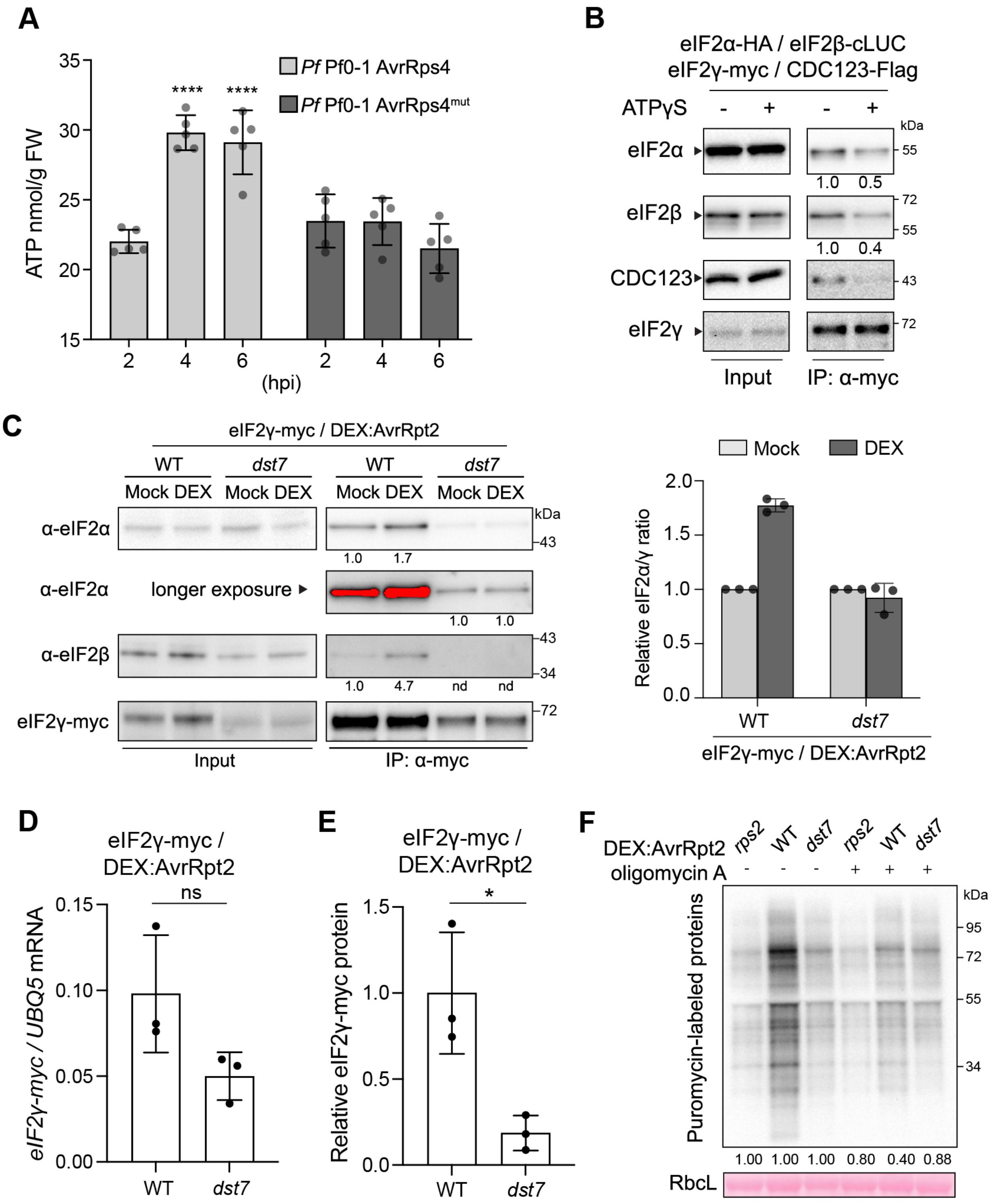

Figure S4. Effects of ATP on assembly of the eIF2 complex. Related to Figure 4.

(A) ATP levels in the Ws-2 ecotype in response to *Pf* Pf0-1/AvrRps4 or *Pf* Pf0-1/AvrRps4^{mut} (n = 5). One-way ANOVA.

(B) Effects of ATPγS on the *in vitro* assembly of the eIF2 complex. Proteins were synthesized using a wheat germ system and incubated with or without 10 mM ATPγS followed by co-IP with anti-myc beads. Numbers below the blot show relative band intensity normalized to IP of eIF2γ-myc.

(C) eIF2 complex assembly in WT or *dst7* plants upon ETI-induction. Samples were collected at 4 hpi with Mock or DEX. Relative band intensity of the immunoblots (left panel) was normalized to IP of eIF2γ-myc (numbers below the blot) and their relative ratios (right panel) are presented as mean ± SD. nd, not detected.

(D and E) mRNA (D) and protein (E) levels of *eIF2γ-myc* normalized to *Ubiquitin 5* (*UBQ5*) and WT, respectively. Data are presented as mean ± SD (n = 3). Two-tailed Student's *t*-test.

(F) New protein synthesis measured by the SUnSET assay of plants expressing *DEX:AvrRpt2* in WT, *rps2* or *dst7* background with or without oligomycin A treatment. All samples were treated with 20 μM DEX for 2.5 hr before application of 20 μM oligomycin A. Numbers show relative lane intensity of the immunoblot normalized to the non-treated sample of each genotype. Ponceau S-stained RbcL was used as a loading control.
